# The effect of small-molecule inhibition of MAPKAPK2 on cell ageing phenotypes of fibroblasts from human Werner syndrome

**DOI:** 10.1186/1752-153X-7-18

**Published:** 2013-01-29

**Authors:** Terence Davis, Michal J Rokicki, Mark C Bagley, David Kipling

**Affiliations:** 1 Institute of Cancer and Genetics, School of Medicine, Cardiff University, Cardiff, CF14 4XN, UK; 2 Department of Chemistry, School of Life Sciences, University of Sussex, Falmer, Brighton, East Sussex, BN1 9QJ, UK

**Keywords:** Werner syndrome, Senescence, p38, MAP kinase, MK2, MAPKAPK2, Stress, Ageing, Signalling

## Abstract

Fibroblasts derived from the progeroid Werner syndrome (WS) show reduced replicative lifespan and a “stressed” morphology, both phenotypes being alleviated by using the p38 MAP kinase inhibitor SB203580. Because p38 is a major hub for the control of stress-signalling pathways we were interested in examining the possible role for downstream kinases in order to refine our understanding of the role of p38 signalling in regulation of WS cell growth. To this end we treated WS and normal fibroblasts with MK2 inhibitors to determine whether MK2 inhibition would affect either the growth or morphology of WS cells. The first inhibitor, 7,8-dihydroxy-2,4-diamino-3-cyanobenzopyranopyridine (inhibitor **2**), resulted in inhibition of WS cell growth and had no effect on morphology, effects that occurred below the level needed to inhibit MK2 and thus suggestive of inhibitor toxicity. The second inhibitor, 2-(2-quinolin-3-ylpyridin-4-yl)-1,5,6,7-tetrahydro-4*H*-pyrrolo-[3,2-*c*]pyridin-4-one (**CMPD16**), resulted in a significant extension of WS fibroblast replicative capacity compared to normal cells. In addition, **CMPD16** reverted the WS cellular morphology to that seen in normal dermal fibroblasts. These data suggest that MK2 activity plays a substantial role in proliferation control in WS cells. **CMPD16** was not as effective in cellular lifespan extension as SB203580, however, suggesting that, although MK2 is a downstream kinase involved in cell cycle arrest, other p38 targets may play a role. Alternatively, as **CMPD16** is toxic to cell growth at levels just above those that extend lifespan, it is possible that the therapeutic window is too small. However, as **CMPD16** does show significant effects in WS fibroblasts, this acts as proof-of-principle for the efforts to design and synthesise improved MK2 inhibitors. As MK2 is involved in inflammatory processes and inflammation plays a major role in WS phenotypes, these data suggest MK2 as a potential therapeutic target for the treatment of Werner syndrome.

## Findings

Werner syndrome (WS) is a genetic disorder where individuals show premature onset of many clinical features of old age and is used as a model to investigate normal ageing processes [1]. The molecular mechanism of WS is related to accelerated cell ageing. Normal human cells divide a limited number of times before entering replicative senescence [2]. This is postulated to contribute to normal human ageing [1] and fibroblasts from WS patients have a much-reduced replicative capacity [3]. The premature senescence of WS cells is thought to be a stress response, and the stress-induced p38 MAP kinase pathway is activated in young WS cells [3]. Treatment with the p38 inhibitor SB203580 increased the growth rate and the cellular life span of primary WS fibroblasts, and rescued their senescent-like morphology [3]. Essentially, SB203580 reverted the phenotypic characteristics of WS fibroblasts, implicating a role for both p38 and stress signalling in WS. These data suggested a possible therapeutic role for p38 inhibitors in WS [4]. However, it has been shown that long-term use of p38 inhibitors in both humans and mice has toxic effects and low therapeutic efficacy [5]. The reasons for this are unknown but may relate to the complexities of the pathways regulated by p38, which is at the hub of the stress-induced response and regulates many downstream kinases and cellular processes [6]. It has thus been suggested that targeting proteins downstream of p38 may be advantageous due to the more limited pathways affected. MAPKAPK2 (MK2) is one particularly attractive target [7] as it is known to regulate the cell cycle [8], is involved in regulating cellular morphology [9], and plays a role in inflammatory processes [10] that are prevalent in WS [4]. In addition, the observation that mice null for MK2 are viable, whereas mice null for p38 are lethal, suggests that therapeutic inhibition of MK2 may prove less problematical than p38 inhibition [10].

Previous work using MK2 inhibitors in WS cells was inconclusive because the inhibitors resulted in cessation of cellular growth that appeared to be unrelated to the ability of the inhibitors to inhibit MK2 [11], suggesting off-target issues (or cellular toxicity) that are a known problem with MK2 inhibitors [7]. In addition, the WS cells used previously were telomerase-immortalised rather than primary cells. Although these telomerase-immortalised cells share many of the aged features of primary WS cells [12], they do show different behaviours in certain situations, as exemplified by the differential effects seen with JNK1/2 inhibitors in these cell types [13].

**Figure 1:**
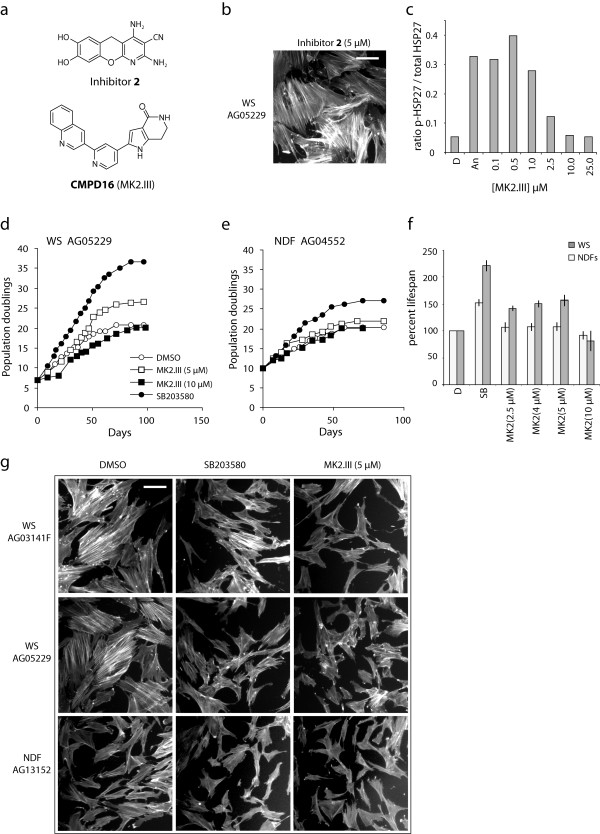
**Effects of MK2 inhibitors of the growth and morphology of WS fibroblasts. **(**a**) Structures of the MK2 inhibitors. (**b**) F-actin stress fibre phenotype of primary AG05229 cells grown in the presence of 5.0 μM inhibitor **2** (a similar phenotype is seen at 2.5 μM). Bar = 100 μm. (**c**) Titration inhibition of MK2 profile by MK2.III as measured by HSP27 phosphorylation ELISA. (**d, e**) Growth of primary WS AG05229 and NDF AG04552 cells in the presence of MK2.III and SB203580 in population doublings (PDs) versus days. (**f**) Histogram illustrating the percentage increase in replicative capacity of WS fibroblasts compared to NDFs for the various treatments with DMSO = 100% lifespan. This data is an average of two strains for both WS (AG05229 and AG03141) and NDFs (AG04552 and AG13152) as illustrated by the error bars thus showing reproduciblity. The lifespan is calculated by taking into account the PDs already attained by the cells prior to arrival from the Coriell cell repository, e.g., for AG05229 the lifespan for 5.0 μM MK2.III treatment is (26.5 PD – 7 PD)/(20.5 PD – 7 PD) = 1.444 = 144.4%. (**g**) Effects of SB203580 or MK2.III on the cellular morphology and F-actin stress fibre phenotype of WS fibroblasts and NDFs. Bar = 100 μm; all panels same magnification.

In this current study, we grew primary WS AG05229 dermal fibroblasts to senescence in the continuous presence of the MK2 inhibitor 7,8-dihydroxy-2,4-diamino-3-cyanobenzopyranopyridine (inhibitor **2** from [11]; see Figure 1a) at 2.5 μM and 5.0 μM (see Additional file 1 for materials and methods). Previously use of this inhibitor at 2.5 μM had no effect on the growth of telomerised WS cells whereas 10 μM was completely inhibitory [11]. Similarly, in this work we found that inhibitor **2** at 2.5 μM had little effect on the replicative capacity of primary WS cells (21 population doublings (PDs) compared to 20.5 PDs for the DMSO control), whereas 5.0 μM was inhibitory, albeit not completely (resulting in a replicative capacity of 16.1 PDs). In addition inhibitor **2** had no effects on the aged morphology of the cells, which retained extensive F-actin stress fibres (Figure 1b; compare to Figure 1g middle left panel). The data with inhibitor **2** suggest that either MK2 inhibition reduces cellular growth, which is inconsistent with the observation that MK2 can promote cell cycle arrest [8], or that inhibitor **2** is having an off-target or toxic effect. Thus, in order to study the role of MK2 in signalling processes in WS cells, novel MK2 inhibitors with better target specificity are required and the pharmaceutical industry is making a major effort in this direction [7].

Recently, the inhibitor 2-(2-quinolin-3-ylpyridin-4-yl)-1,5,6,7-tetrahydro-4*H*-pyrrolo-[3,2-*c*pyridin-4-one (**CMPD16** of [14]; see Figure 1a) became commercially available (designated as MK2.III). This inhibitor is promising due to its potency and good target specificity [14]. MK2.III prevented anisomycin induced HSP27 phosphorylation in human fibroblasts with an IC_50_ below 2.5 μM, which is in good agreement with the published IC_50_ seen in U937 cells [14], and showed maximal inhibition at 10 μM (Figure 1c). We thus grew two primary normal dermal fibroblast (NDF) and WS fibroblast strains in the presence of MK2.III at various concentrations (2.5, 4.0, 5.0 and 10 μM) and compared the replicative capacity with untreated cells and with cells grown in the presence of 2.5 μM SB203580. The primary normal fibroblast strains chosen were from elderly individuals and have replicative capacities in the same range as seen in the WS fibroblasts [15]. MK2.III treatment at 5.0 μM had the greatest effect on the replicative capacity of WS cells compared to untreated cells (illustrated for WS strain AG05229 where the replicative capacity increased from 20.5 PDs to 26.5 PDs: Figure 1d). The percentage increase in experimental replicative capacity using MK2.III at 5.0 μM for the two WS strains compared to untreated cells of 50.3 ± 5.6% was statistically significant (Figure 1f; Table 1). In contrast MK2 inhibition had little effect on NDFs (21.9 PDs compared to 20.5 PDs at 5.0 μM for NDF strain AG04552: Figure 1e). For the NDF strains, MK2.III resulted in a statistically insignificant increase in replicative capacity of 8.0 ± 7.0% (Figure 1f; Table 1). In addition, the increase in replicative capacity by MK2.III was significantly greater in WS fibroblasts compared to NDFs (Table 1). MK2.III also increased replicative capacity at 2.5 and 4.0 μM, but not to the degree seen using 5.0 μM, whereas 10 μM was inhibitory (Figure 1d-f). In contrast SB203580 resulted in much greater increases in replicative capacity for both NDFs and WS fibroblasts (Figure 1d-f), in agreement with previous reports for these cells [3,15]. The increase in replicative capacity using SB203580 is significantly greater for WS fibroblasts than for NDFs (Table 1), again consistent with previous data.

**Table 1:** Statistical tests

**Treatment**	**NDFs**^**a**^	**WS**^**a**^	**NDFs / WS**^**b**^
MK2.III (5μM)	*p* > 0.37	*p* < 0.05	*p* < 0.029
SB203580 (2.5μM)	*p* < 0.05	*p* < 0.036	*p* < 0.014

^a^ Probability that inhibitor treatment results in no significant extension of replicative capacity for NDFs or WS fibroblasts compared to DMSO-treated cells (paired *t*-test).

^b^ Probability that the extension of replicative capacity seen for WS cells is not greater than that seen for NDFs for same treatment (unpaired 2-tailed *t*-test).

One of the defining features of WS fibroblasts is their aged morphology and extensive F-actin stress fibre formation even at low PD levels, and p38 inhibition using SB203580 treatment reverted this morphology to that seen in low PD NDFs ([3]; see Figure 1g). As F-actin stress fibre formation is associated with HSP27 phosphorylation by MK2 [9], we examined this phenotype in cells treated with MK2 inhibitors. For both WS strains, many control cells were enlarged with extensive F-actin stress fibres (Figure 1g). Continuous treatment with 5.0 μM MK2.III reverted this morphology, with the bulk of treated cells being smaller and with few F-actin stress fibres visible. With regard to this phenotype MK2.III appeared to be more effective than SB203580, particularly in WS strain AG03141F (Figure 1g). In contrast very few enlarged cells with F-actin stress fibres were seen in NDFs, and they were unaffected by treatment with either SB203580 or MK2.III.

These data suggest that the accelerated senescence seen in WS fibroblasts is due, at least partially, to activation of MK2. However, MK2.III is less effective than the p38 inhibitor SB203580 at extending replicative capacity. There are several possible reasons for this difference: firstly, SB203580 may target more than one kinase involved in cell cycle arrest [16]; secondly, the stress-induced growth arrest via p38 may operate though multiple p38 target proteins; thirdly, it may be that the therapeutic window for MK2.III is too small, since it soon becomes inhibitory to cellular growth above its optimal concentration. As our previous work suggests that, of the known candidates for SB203580, p38 is the primary target for its effects in WS cells [13,17], and MK2.III is as effective as SB203580 at reverting the cellular morphology phenotype, it is possible that p38 inhibits cell growth via multiple targets. Possible p38 effectors include the MNKs and MSKs that are activated in response to environmental stresses, the latter in a manner inhibited by SB203580 [6] with the MSKs not being inhibited by MK2.III [14]. In addition there are several transcription factors that are p38 targets [6]. The detailed p38 pathways and their effects on cellular proliferation are not fully understood. It is important to note that MK2.III does not have its effects by actually inhibiting p38 [14]. However, because the possibility of a small therapeutic window for MK2.III remains, work is in progress to synthesise or otherwise obtain other MK2 inhibitors with different properties to extend this work. Overall, this work provides support for the role of MK2 in accelerated cell senescence in WS fibroblasts, and the possible therapeutic targeting of MK2 in WS individuals, and thus supports further work in this area when suitable MK2 inhibitors become available.

## Description of additional material

Methods: word file documenting materials and methods used in this work.

## Competing interests

The authors declare that they have no competing interests.

## Authors’ contributions

TD participated in study design and coordination, manuscript preparation, growth experiments and phalloidin assays, and helped conceive the study, MJR performed the ELISA assays, MCB participated in study design, synthesis of MK2 inhibitors, manuscript preparation and helped conceive the study, DK conceived the study, participated in its design and coordination and helped to draft the manuscript. All authors read and approved the final manuscript.

## Supplementary Material

Additional file 1 Materials and Methods. Click here for file
