# Supplementary material for: The effect of small-molecule inhibition of MAPKAPK2 on cell ageing phenotypes of fibroblasts from human Werner syndrome
Source: Chem Cent J. 2013 Jan 29;7:18. doi: 10.1186/1752-153X-7-18 (PMC3562269; doi:10.1186/1752-153X-7-18)
Supplement: Additional file 1 — Materials and Methods. [file 1752-153X-7-18-S1.doc]

**Additional Material**

**Materials and Methods**

1. Materials. Werner syndrome cell strains AG05229 and AG03141 and NDF strains AG04552 and AG13152 were obtained from Coriell Cell Repositories (Camden, NJ, USA). The NDFs were from individuals of 64 and 80 yrs of age respectively. The telomerised HCA2 cells have been described previously [1]. The MK2 inhibitor MK2.III was obtained from Merck, UK. Inhibitor **2** was synthesised according to [2]. Anisomycin was purchased from Tocris Chemical Co. (Bristol, UK).

2. Determination of the ability of inhibitors to inhibit the p38 pathway.

The ability to inhibit the p38 stress-signalling pathway was tested in human hTERT-immortalised HCA2 dermal cells [1] using an ELISA system (Cell Signalling, NEB, UK). Cells were seeded in 100 mm dishes in Earle’s modification of Eagle medium (EMEM) and incubated at 37 °C for 48 h as described previously [3]. The medium was supplemented with inhibitor MK2.III (**CMPD16)** dissolved in DMSO at final concentrations from 100 nM to 25 µM and the cells incubated for a further 2 h. Then anisomycin was added to the medium at 30 µM and the cells harvested 45 min later. Samples using DMSO only, and DMSO plus anisomycin, were used as controls. Cells were harvested, proteins isolated and ELISA assays were carried out according to the manufacturer’s instructions. Kinase activity was detected using antibodies specific for the phosphorylated form of HSP27, and antibodies that detect the total levels of HSP27; the degree of activation was measured as the ratio of phospho-protein/total protein. In this system anisomycin activates activates p38, which results in MK2 phosphorylation and subsequent phosphorylation of the small heat shock protein HSP27. As MK2 is the major HSP27 kinase, the activity of p38 can be assessed by the phosphorylation status of HSP27.

3. Investigating the effects of MK2 inhibitors on WS cell growth.

Cells were grown in Earles’s modified Eagle medium as previously described [3]. Population doublings (PD) were calculated according to the formula: PD = log(*N*_t_/*N*_o_)/log2, where *N*_t_ is number of cells counted and *N*_o_ is number of cells seeded. For drug treatments the culture medium was supplemented with inhibitors at various concentrations dissolved in DMSO, with the medium being replaced daily. For controls an equivalent volume of the drug solvent (DMSO), or SB203580 at 2.5 µM, was added to the medium.

4. Immunofluorescence microscopy

Actin staining for immunofluorescence microscopy was performed essentially as described previously [3]. Briefly, the cells were plated into 35 mm plastic dishes in EMEM and allowed to settle for 48 hours. The cells were then washed in phosphate buffered saline (PBS), fixed in 3.7% paraformaldehyde for 20 mins and permeabilised with 0.1% Triton-X100 for 20 mins. F-actin was detected using fluorescein isothiocyanate-conjugated phalloidin (33 µg/ml), diluted 1:50 in PBS for 30 mins in the dark, followed by washing in PBS.

1. Bagley MC, Davis T, Dix MC, Rokicki MJ, Kipling D: **Rapid synthesis of VX-745: p38 MAP kinase inhibition in Werner syndrome cells.** *Bioorg Med Chem Lett* 2007, **17:**5107-5110.

2. Davis T, Bagley MC, Dix MC, Murziani PG, Rokicki MJ, Widdowson CS, Zayed JM, Bachler MA, Kipling D: **Synthesis and in vivo activity of MK2 and MK2 substrate-selective p38α(MAPK) inhibitors in Werner syndrome cells.** *Bioorg Med Chem Lett* 2007, **17:**6832-6835.

3. Davis T, Baird DM, Haughton MF, Jones CJ, Kipling D: **Prevention of accelerated cell aging in Werner syndrome using a p38 mitogen-activated protein kinase inhibitor.** *J Gerontol A Biol Sci Med Sci* 2005, **60:**1386-1393.
